# A General and Simple Diastereoselective Reduction by l-Selectride: Efficient Synthesis of Protected (4*S*,5*S*)-Dihydroxy Amides

**DOI:** 10.3390/molecules15042771

**Published:** 2010-04-16

**Authors:** Bo Yin, Dong-Nai Ye, Kai-Hui Yu, Liang-Xian Liu

**Affiliations:** Department of Chemistry and Biology, Ganan Normal University, Ganzhou, Jiangxi 341000, China

**Keywords:** l-Selectride, 3-hydroxyglutarimide, (4*S*,5*S*)-dihydroxyamide

## Abstract

A general approach to (4*S*,5*S*)-4-benzyloxy-5-hydroxy-*N*-(4-methoxybenzyl) amides **10** based on a diastereoselective reduction of (5*S*,6*RS*)-6-alkyl-5-benzyloxy-6-hydroxy-2-piperidinones **6** and their tautomeric ring-opened keto amides **7** is described. The reduction with l-Selectride at -20 °C to room temperature afforded the products **10** in excellent yields and moderate to high *syn*-diastereoselectivities.

## 1. Introduction 

The (4,5)-dihydroxycarboxylate moiety is a critical framework shared by many bioactive compounds, such as Microcarpalide (**1**), which is a 10-membered lactone that was isolated from the fermentation broth of an unidentified endophytic fungus by Hemscheidt and co-workers in 2001 [1], and Kalanchosine dimalate (KMC, **2)** [2], which is an anti-inflammatory salt from the fresh juice of the aerial parts of *Kalanchoe brasiliensis*, as well as natural gastroprotective 3,4-dihydroisocoumarins, such as amicoumacin C (**3**) [3,4] and AI-77B (**4**) [5,6]. Both the stereochemical variation at C-4, C-5 and the interesting biological activities exhibited by these compounds make them attractive synthetic targets [1,5,6,7]. A number of methods have been developed for the synthesis of these compounds [8,9,10,11,12,13], but few methods for the construction of the (4,5)-dihydroxycarboxylate moiety [14,15,16,17,18]. Generally, chiral pool starting materials or Sharpless asymmetric dihydroxylation was used in the construction of the (4,5)-dihydroxycarboxylate moiety.

**Figure 1:**
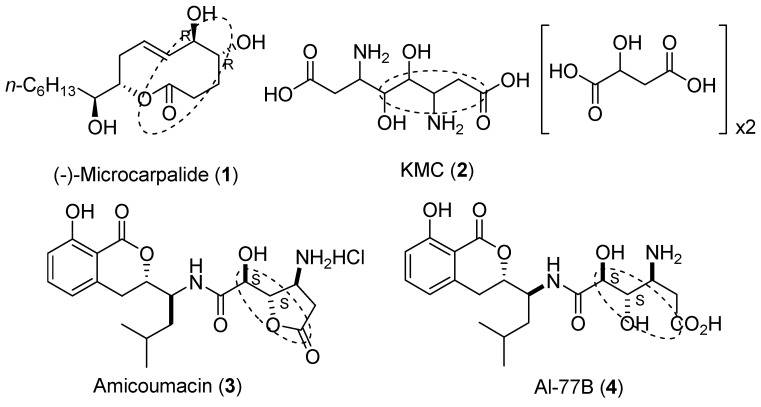
(4,5)-Dihydroxycarboxylate derivatives.

**Scheme 1:**
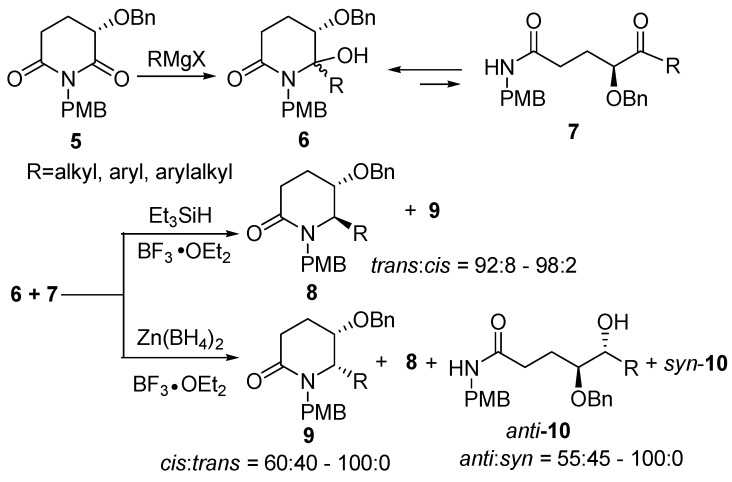
The synthesis of 6-alkyl-5-benzyloxy-2-piperidinones.

Previously, we have shown that the protected (*S*)-3-hydroxyglutarimide **5** may serve as a versatile building block for the asymmetric synthesis of a variety of 2,6-disubstituted 3-hydroxypiperidines [19,20,21,22,23]. A flexible regio- and diastereoselective reductive alkylation method was developed for the conversion of **5** to *trans*-6-alkyl-5-benzyloxy-2-piperidinone derivatives **8** [20]**.** Recently, we also developed a chemo- and diastereoselective transformation of the *N*,*O*-acetals **6** and their chain tautomers **7**, readily derived from protected 3-hydroxyglutarimide **5**, into cyclic products (5*S*,6*S*/*R*)-6-alkyl-5-benzyloxy-2-piperidinones **9**/**8**, and *anti*-**10***/syn*-**10** with a combination of boron trifluoride etherate/zinc borohydride in modest chemo- and diastereoselectivities (Scheme **1**) [24]. Moreover, the reduction with zinc borohydride in the absence of BF_3_•OEt_2_ leading exclusively to the formation of the ring-opening products *anti***-10** in excellent *anti*-diastereoselectivities was exploited. In addition, we reported the application of this new variation to the asymmetric synthesis of (+)-azimic acid [25]. 

**Scheme 2:**
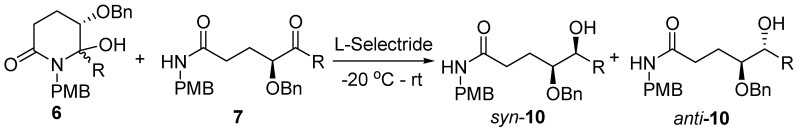
The diastereoselective reduction by l-Selectride.

In the continuation of our interest in the amino acid chiral template-assisted synthesis of natural and unnatural bioactive compounds, as a part of our research program aimed at developing enantioselective syntheses of naturally occurring bioactive compounds, such as Microcarpalide (**1**), we decided to explore the construction of the (4,5)-dihydroxycarboxylate moiety in order to develop a simple and feasible approach to *syn*-**10**, a key intermediate (R = CH=CH_2_) for the synthesis of **1**. Herein we report a diastereoselective reduction of **6** and **7** employing l-Selectride as the reductive agent to obtain *syn*-**10** (Scheme **2**). 

## 2. Results and Discussion 

The requisite 6-alkyl-5-benzyloxy-6-hydroxy-2-piperidinones **6**, together with their ring-opened keto amide tautomers **7**, were prepared *via* the addition of Grignard reagents to (*S*)-3-benzyloxy-glutarimide **5** under our recently improved conditions [23]. Treatment of the tautomeric mixture of **6a** and **7a** with 1.2 molar equiv of l-Selectride in THF (−20 °C - rt) yielded *syn*-**10a** and *anti*-**10a** in a ratio of 86:14 (combined yield: 93%). To explore the generality of the process, a series of hemi-azaketals **6** and their opened keto amide tautomers **7** were investigated using l-Selectride as reductive agent [26,27,28,29], and the results are reported in Table **1**. 

**Table 1:** Results of reduction according to the procedure shown in Scheme **2**.

Entry	R	Yield [%] *^a^*	*syn*/*anti* ratio
1	CH_3_ (**10a**)	93	6:1 *^b^*
2	C_2_H_5_ (**10b**)	97	7:1 *^c^*
3	*n*-C_4_H_9_ (**10c**)	97	7:1 *^c^*
4	*n*-C_5_H_11_ (**10d**)	95	23:2 *^c^*
5	*n*-C_8_H_17_ (**10e**)	98	23:2 *^b^*
6	*n*-C_12_H_25_ (**10f**)	85	9:1 *^b^*
7	*n*-C_16_H_33_ (**10g**)	83	7:1 *^b^*
8	*i*-Bu (**10h**)	92	3:1 *^c^*
9	Ph (**10i**)	81	3:1 *^b^*
10	Bn (**10j**)	92	11:2 *^c^*
11	PhCH_2_CH_2_ (**10k**)	82	7:2 *^c^*

*^a^* Isolated yield of **10** starting from **6** and **7**. *^b^* Ratio determined by ^1^H-NMR analysis. *^c^* Ratio based on HPLC analysis.

**Scheme 3:**
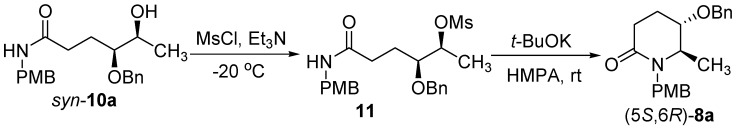
The synthesis of (5*S*,6*R* )-6-methyl-5-benzyloxy-2-piperidinones.

As can be seen from Table 1, high yields and modest to high *syn*-selectivities were obtained for all hemi-azaketals tested. It is interesting to note that modest *syn*-selectivities were obtained in the case where **6** and **7** bearing *i*-Bu or Ph (Table **1****,** entries 8 and 9) as well as PhCH_2_CH_2_ (Table **1****,** entry 11). The stereochemistry of the major diastereomer **10** was assigned to *syn*-conformer according to the observed vicinal coupling constants [24] (*J*_4,5_ = 5.1 Hz for *syn*-**10a** and *J*_4,5_ = 4.5 Hz for *anti*-**10a**; *J*_4,5_ = 5.2 Hz for *syn*-**10b** and *J*_4,5_ = 4.3 Hz for *anti*-**10b**; *J*_4,5_ = 5.1 Hz for *syn*-**10c** and *J*_4,5_ = 4.2 Hz for *anti*-**10c**; *J*_4,5_ = 5.1 Hz for *syn*-**10e** and *J*_4,5_ = 4.4 Hz for *anti*-**10e**; *J*_4,5_ = 5.1 Hz for *syn*-**10g** and *J*_4,5_ = 4.5 Hz for *anti*-**10g**; *J*_4,5_ = 6.1 Hz for *syn*-**10i** and *J*_4,5_ = 5.1 Hz for *anti*-**10i**). In addition, the stereochemistry of diastereomers *syn*-**10** was confirmed by converting *syn***-10** to (5*S*,6*R*)-6-alkyl-5-benzyloxy-2-piperidin-ones **8**. For example, *syn***-10a** can be converted to *anti*-**8a** in 78% yield by mesylation (MsCl, Et_3_N, CH_2_Cl_2_, −20 °C, 1 h) and *t*-BuOK-promoted cyclization (HMPA, THF, rt, 24 h) (Scheme **3**). 

**Figure 2:**
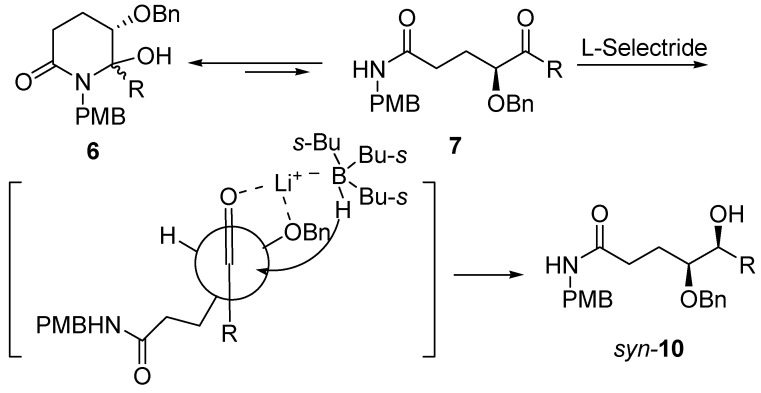
A plausible Cram chelation-controlled pathway for the *syn*-diastereoselective formation of *syn-***10**.

The fact that starting from the tautomeric mixture of **6** and **7** *syn*-diastereomer **10** was obtained in modest to high diastereoselectivity is in accordance with a Cram model-based mechanism [30,31,32,33,34]. It was envisioned that the hydride to approach C-5 carbon from the same side of the chelate C-4 benzyloxy substituent led to the formation of *syn*-isomer because of the chelation between lithium ion and oxygen atom of the C-4 oxygen as well as C-5 carbonyl oxygen (Figure **2**), which not only switches the equilibrium towards **7**, but also allows the reduction to undergo with a Cram chelation-controlled manner. 

## 3. Conclusions 

In summary, a simple and efficient route to protected (4S,5S)-dihydroxy amides *via* the reduction of the tautomeric mixture of **6** and **7** with l-Selectride has been developed. This strategy offers a concise platform for the construction of (4*S*,5*S*)-dihydroxycarboxylate moieties under mild conditions. As such, this method is complementary, in part, to our previously established *anti*-diastereoselective method. 

## 4. Experimental 

### 4.1. General methods 

Melting points were determined on a Yanaco MP-500 micro melting point apparatus and are uncorrected. Infrared spectra were measured with a Nicolet Avatar 360 FT-IR spectrometer using film KBr pellet technique. ^1^H-NMR spectra were recorded in CDCl_3_ on a Bruker 400 or a Varian unity +500 spectrometer with tetramethylsilane as an internal standard. Chemical shifts are expressed in δ (ppm) units downfield from TMS. Mass spectra were recorded with Bruker Dalton Esquire 3000 plus LC-MS apparatus. Optical rotations were measured with a Perkin-Elmer 341 automatic polarimeter. Elemental analysis was carried out on a Perkin-Elmer 240B instrument. Flash column chromatography was carried out with silica gel (300-400 mesh). THF was distilled over sodium and CH_2_Cl_2_ was distilled over P_2_O_5_ under N_2_. 

### 4.2. General procedure for preparation of syn-**10**

To a cooled (−20 °C) solution of tautomeric mixture **6/7** [20] (1.0 mol equiv) in THF (0.1 M) was added dropwise a solution of l-Selectride (1.2 mol equiv) under argon atmosphere and the mixture was stirred at −20 ~ −10 °C for 1 h. Then, the mixture was allowed to slowly warm to room temperature and was stirred at room temperature overnight. The reaction was quenched with a saturated aqueous NH_4_Cl. After extraction with CH_2_Cl_2_, the combined organic layers were washed with brine, dried over anhydrous Na_2_SO_4_, filtered, and concentrated under reduced pressure. The residue was purified by flash chromatography on silica gel (eluent: EtOAc/Petroleum ether = 1:2), some pure *syn*-**10** and the mixture of *syn*-**10** and *anti*-**10** were obtained. 

*(4S,5S)-4-Benzyloxy-5-hydroxy-N-(4-methoxybenzyl)hexanoyl amide* (*syn*-**10a**): White solid, mp: 74-75 °C; [α]^25^_D_: +4.75 (*c* 1.0, CHCl_3_); IR (film) νmax: 3407, 3305, 1649, 1513, 1248 cm^-1^; ^1^H-NMR (400 MHz, CDCl_3_): δ 7.33-7.25 (m, 5H, Ar-H), 7.15 (d,* J* = 8.6 Hz, 2H, Ar-H), 6.83 (d, *J* = 8.6 Hz, 2H, Ar-H), 5.60 (s, 1H, NH), 4.59 (d, *J* = 11.5 Hz, 1H, OCH_2_), 4.53 (d, *J* = 11.5 Hz, 1H, OCH_2_), 4.32 (dd, *J* = 14.5, 5.6 Hz, 1H, NCH_2_), 4.27 (dd, *J* = 14.5, 5.6 Hz, 1H, NCH_2_), 3.78 (s, 3H, OCH_3_), 3.70 (m, 1H, H-4), 3.35 (dd,* J* = 6.4, 5.1 Hz, 1H, H-5), 2.59 (d, *J* = 2.8 Hz, 1H, OH), 2.25 (t, *J* = 7.4 Hz, 2H, H-2), 2.04 (ddd, *J* = 14.0, 7.4, 4.9 Hz, 1H, H-6), 1.82 (ddd, *J* = 14.0, 7.4, 6.8 Hz, 1H, H-3), 1.17 (t, *J* = 6.4 Hz, 3H, CH_3_); ^13^C-NMR (100 MHz, CDCl_3_): δ 172.4 (C=O), 158.9, 138.2, 130.3, 129.1 (2×C), 128.4 (2×C), 127.9 (2×C), 127.8, 114.0 (2×C), 82.1 (C-5), 71.9 (C-4), 68.6 (OCH_2_), 55.2 (OCH_3_), 43.0 (NCH_2_), 31.6, 25.6, 18.9; MS (ESI): 358 [M+H]^+^, 380 [M+Na]^+^; Anal calcd for C_21_H_27_NO_4_: C, 70.56; H, 7.61; N, 3.92. Found C, 70.31; H, 7.76; N, 4.25. 

*(4S,5S)-4-Benzyloxy-5-hydroxy-N-(4-methoxybenzyl)heptanoyl amide* (*syn*-**10b**): White solid, mp: 122-124 °C; [α]^25^_D_: +1.86 (*c* 1.2, CHCl_3_); IR (film) ν_max_: 3407, 3306, 1649, 1513, 1248 cm^-1^; ^1^H-NMR (400 MHz, CDCl_3_): δ 7.32-7.25 (m, 5H, Ar-H), 7.15 (d, *J* = 8.7 Hz, 2H, Ar-H), 6.83 (d, *J* = 8.7 Hz, 2H, Ar-H), 5.57 (s, 1H, NH), 4.59 (d, *J* = 11.5 Hz, 1H, OCH_2_), 4.53 (d, *J* = 11.5 Hz, 1H, OCH_2_), 4.32 (dd, *J* = 14.4, 5.5 Hz, 1H, NCH_2_), 4.28 (dd, *J* = 14.5, 5.5 Hz, 1H, NCH_2_), 3.78 (s, 3H, OCH_3_), 3.43 (m, 1H, H-4), 3.36 (ddd, *J* = 5.6, 5.6, 5.2 Hz, 1H, H-5), 2.42 (s, 1H, OH), 2.26 (t, *J* = 7.4 Hz, 2H, H-3), 2.03 (ddd, *J* = 13.8, 7.1, 5.2 Hz, 1H, H-2), 1.87 (ddd, *J* = 13.8, 7.5, 7.2 Hz, 1H, H-2), 1.55 (ddd, *J* = 13.8, 7.5, 4.1 Hz, 1H, H-6), 1.46 (ddd, *J* = 13.8, 7.2, 5.2 Hz, 1H, H-6), 0.95 (t, *J* = 7.5 Hz, 3H, CH_3_); ^13^C-NMR (100 MHz, CDCl_3_): δ 172.4 (C=O), 158.9, 138.2, 130.4, 129.1 (2×C), 128.4 (2×C), 127.8 (2×C), 127.7, 114.0 (2×C), 80.8 (C-5), 74.0 (C-4), 72.5 (OCH_2_), 55.2 (OCH_3_), 43.0 (NCH_2_), 31.8, 26.2, 25.9, 10.2; MS (ESI): 371 [M+H]^+^, 394 [M+Na]^+^, 410 [M+K]^+^; Anal calcd for C_22_H_29_NO_4_: C, 71.13; H, 7.87; N, 3.77. Found C, 71.03; H, 7.55; N, 3.71. 

*(4S,5S)-4-Benzyloxy-5-hydroxy-N-(4-methoxybenzyl)nonanoyl amide* (*syn*-**10c**): Waxy solid; [α]^25^_D_: +1.90 (*c* 1.5, CHCl_3_); IR (film) ν_max_: 3407, 3305, 1650, 1513, 1248 cm^-1^; ^1^H-NMR (500 MHz, CDCl_3_): δ 7.35-7.25 (m, 5H, Ar-H), 7.16 (d, *J* = 8.7 Hz, 2H, Ar-H), 6.84 (d, *J* = 8.7 Hz, 2H, Ar-H), 5.55 (s, 1H, NH), 4.60 (d, *J* = 11.5 Hz, 1H, OCH_2_), 4.53 (d, *J* = 11.5 Hz, 1H, OCH_2_), 4.33 (dd, *J* = 14.4, 5.6 Hz, 1H, NCH_2_), 4.28 (dd, *J* = 14.4, 5.6 Hz, 1H, NCH_2_), 3.78 (s, 3H, OCH_3_), 3.52 (m, 1H, H-4), 3.36 (ddd, *J* = 6.2, 5.1, 5.1 Hz, 1H, H-5), 2.33 (s, 1H, OH), 2.26 (t, *J* = 7.4 Hz, 2H, H-2), 2.05 (ddd, *J* = 14.0, 7.4, 5.2 Hz, 1H, H-3), 1.87 (ddd, *J* = 14.0, 7.6, 7.4 Hz, 1H, H-3), 1.52-1.40 (m, 3H), 1.36-1.25 (m, 3H), 0.89 (t, *J* = 7.1 Hz, 3H, CH_3_); ^13^C-NMR (125 MHz, CDCl_3_): δ 172.4 (C=O), 159.0, 138.2, 130.3, 129.2 (2×C), 128.4 (2×C), 127.9 (2×C), 127.8, 114.0 (2×C), 81.1 (C-5), 72.6 (C-4), 72.5 (OCH_2_), 55.3 (OCH_3_), 43.1 (NCH_2_), 33.1, 31.8, 27.9, 23.9, 22.7, 14.0; MS (ESI): 400 [M+H]^+^, 422 [M+Na]^+^, 438 [M+K]^+^; Anal calcd for C_24_H_33_NO_4_: C, 72.15; H, 8.33; N, 3.51. Found C, 72.34; H, 8.36; N, 3.66. 

*(4S,5S)-4-Benzyloxy-5-hydroxy-N-(4-methoxybenzyl)decanoyl amide* (*syn*-**10d**): Waxy solid; [α]^25^_D_: ‑1.76 (*c* 2.3, CHCl_3_); IR (film) ν_max_: 3411, 3304, 2931, 1646, 1513, 1248 cm^-1^; ^1^H-NMR (400 MHz, CDCl_3_): δ 7.32-7.26 (m, 5H, Ar-H), 7.15 (d, *J* = 8.5 Hz, 2H, Ar-H), 6.82 (d, *J* = 8.5 Hz, 2H, Ar-H), 5.85 (s, 1H, NH), 4.59 (d, *J* = 11.5 Hz, 1H, OCH_2_), 4.52 (d, *J* = 11.5 Hz, 1H, OCH_2_), 4.32 (dd, *J* = 14.5, 5.6 Hz, 1H, NCH_2_), 4.28 (dd, *J* = 14.5, 5.6 Hz, 1H, NCH_2_), 3.78 (s, 3H, OCH_3_), 3.52 (m, 1H, H-4), 3.34 (ddd, *J* = 5.4, 5.4, 5.2 Hz, 1H, H-5), 2.38 (d, *J* = 4.3 Hz, 1H, OH), 2.25 (t, *J* = 7.3 Hz, 2H, H-2), 2.04 (ddd, *J* = 14.0, 7.7, 7.3 Hz, 1H, H-3), 1.87 (ddd, *J* = 14.0, 7.3, 7.1 Hz, 1H, H-3), 1.50-1.40 (m, 3H), 1.35-1.20 (m, 5H), 0.88 (t, *J* = 6.8 Hz, 3H, CH_3_); ^13^C-NMR (100 MHz, CDCl_3_): δ 172.4 (C=O), 159.0, 138.2, 130.4, 129.1 (2×C), 128.4 (2×C), 127.9 (2×C), 127.8, 114.0 (2×C), 81.1 (C-5), 72.6 (C-4), 72.5 (OCH_2_), 55.2 (OCH_3_), 43.0 (NCH_2_), 33.3, 31.8, 26.0, 25.4 (2×C), 22.6, 14.0; MS (ESI): 414 [M+H]^+^, 436 [M+Na]^+^; Anal calcd for C_25_H_35_NO_4_: C, 72.61; H, 8.53; N, 3.39. Found C, 72.33; H, 8.52; N, 3.42. 

*(4S,5S)-4-Benzyloxy-5-hydroxy-N-(4-methoxybenzyl)tridecanoyl amide* (*syn*-**10e**): Waxy solid; [α]^25^_D_: ‑2.21 (*c* 2.3, CHCl_3_); IR (film) ν_max_: 3406, 3304, 2926, 2854, 1646, 1513, 1249 cm^-1^; ^1^H-NMR (400 MHz, CDCl_3_): δ 7.34-7.25 (m, 5H, Ar-H), 7.15 (m, 2H, Ar-H), 6.84 (m, 2H, Ar-H), 5.75 (s, 1H, NH), 4.60 (d, *J* = 11.5 Hz, 1H, OCH_2_), 4.53 (d, *J* = 11.5 Hz, 1H, OCH_2_), 4.33 (dd, *J* = 14.4, 5.6 Hz, 1H, NCH_2_), 4.29 (dd, *J* = 14.4, 5.6 Hz, 1H, NCH_2_), 3.78 (s, 3H, OCH_3_), 3.51 (m, 1H, H-4), 3.36 (ddd, *J* = 5.6, 5.6, 5.1 Hz, 1H, H-5), 2.32-2.23 (m, 2H), 2.26 (s, 1H, OH), 2.05 (m, 1H), 1.88 (ddd, *J* = 14.2, 6.7, 6.7 Hz, 1H), 1.52-1.40 (m, 3H), 1.34-1.20 (m, 11H), 0.88 (t, *J* = 6.9 Hz, 3H, CH_3_); ^13^C-NMR (100 MHz, CDCl_3_): δ 172.4 (C=O), 159.0, 138.3, 130.4, 129.2 (2×C), 128.4 (2×C), 127.9 (2×C), 127.8, 114.0 (2×C), 81.2 (C-5), 72.7 (C-4), 72.6 (OCH_2_), 55.3 (OCH_3_), 43.1 (NCH_2_), 33.4, 31.8 (2×C), 29.7, 29.5, 29.3, 26.0, 25.8, 22.6, 14.1; MS (ESI): 456 [M+H]^+^; Anal calcd for C_28_H_41_NO_4_: C, 73.85; H, 9.01; N, 3.08. Found C, 73.59; H, 8.98; N, 3.06. 

*(4S,5S)-4-Benzyloxy-5-hydroxy-N-(4-methoxybenzyl)heptadecanoyl amide* (*syn*-**10f**): White solid, mp: 68-70 °C; [α]^25^_D_: -2.63 (*c* 1.1, CHCl_3_); IR (film) ν_max_: 3423, 3305, 2924, 2853, 1643, 1513, 1248 cm^-1^; ^1^H-NMR (400 MHz, CDCl_3_): δ 7.35-7.23 (m, 5H, Ar-H), 7.16 (d, *J* = 8.2 Hz, 2H, Ar-H), 6.85 (d, *J* = 8.2 Hz, 2H, Ar-H), 5.70 (s, 1H, NH), 4.61 (d, *J* = 11.5 Hz, 1H, OCH_2_), 4.54 (d, *J* = 11.5 Hz, 1H, OCH_2_), 4.34 (dd, *J* = 14.5, 5.5 Hz, 1H, NCH_2_), 4.30 (dd, *J* = 14.5, 5.5 Hz, 1H, NCH_2_), 3.79 (s, 3H, OCH_3_), 3.52 (m, 1H, H-4), 3.36 (ddd, *J* = 5.3, 5.3, 5.1 Hz, 1H, H-5), 2.30-2.23 (m, 2H), 2.26 (s, 1H, OH), 2.05 (ddd, *J* = 14.0, 7.3, 7.3 Hz, 1H, H-2), 1.88 (ddd, *J* = 14.0, 7.0, 7.0 Hz, 1H, H-2), 1.52-1.40 (m, 3H), 1.35-1.20 (m, 19H), 0.88 (t, *J* = 6.6 Hz, 3H, CH_3_); ^13^C-NMR (100 MHz, CDCl_3_): δ 172.4 (C=O), 159.1, 138.3, 130.4, 129.2 (2×C), 128.5 (2×C), 127.9 (2×C), 127.8, 114.1 (2×C), 81.2 (C-5), 72.7 (C-4), 72.6 (OCH_2_), 55.3 (OCH_3_), 43.1 (NCH_2_), 33.5, 31.9, 31.8, 29.7 (6×C), 29.4, 26.0, 25.8, 22.7, 14.1; MS (ESI): 512 [M+H]^+^, 534 [M+Na]^+^; Anal calcd for C_32_H_49_NO_4_: C, 75.11; H, 9.65; N, 2.74. Found C, 75.39; H, 9.91; N, 2.86. 

*(4S,5S)-4-Benzyloxy-5-hydroxy-N-(4-methoxybenzyl)heneicosanoyl amide* (*syn*-**10g**): White solid, mp: 62-64 °C; [α]^25^_D_: -1.93 (*c* 1.1, CHCl_3_); IR (film) ν_max_: 3419, 3302, 2923, 2852, 1655, 1513, 1249 cm^-1^; ^1^H-NMR (400 MHz, CDCl_3_): δ 7.33-7.27 (m, 5H, Ar-H), 7.17 (d, *J* = 8.6 Hz, 2H, Ar-H), 6.85 (d, *J* = 8.6 Hz, 2H, Ar-H), 5.65 (s, 1H, NH), 4.60 (d, *J* = 11.5 Hz, 1H, OCH_2_), 4.54 (d, *J* = 11.5 Hz, 1H, OCH_2_), 4.33 (dd, *J* = 14.4, 5.5 Hz, 1H, NCH_2_), 4.30 (dd, *J* = 14.4, 5.5 Hz, 1H, NCH_2_), 3.80 (s, 3H, OCH_3_), 3.52 (m, 1H, H-4), 3.35 (m, 1H, H-5), 2.30-2.22 (br s, 1H, OH), 2.26 (t, *J* = 7.4 Hz, 2H, H-2), 2.05 (ddd, *J* = 14.1, 7.4, 7.4 Hz, 1H, H-3), 1.88 (ddd, *J* = 14.1, 7.4, 6.8 Hz, 1H, H-3), 1.52-1.40 (m, 3H), 1.35-1.20 (m, 27H), 0.88 (t, *J* = 6.8 Hz, 3H, CH_3_); ^13^C-NMR (100 MHz, CDCl_3_): δ 172.4 (C=O), 159.1, 138.3, 130.4, 129.2 (2×C), 128.5 (2×C), 127.9 (2×C), 127.8, 114.1 (2×C), 81.2 (C-5), 72.8 (C-4), 72.6 (OCH_2_), 55.3 (OCH_3_), 43.1 (NCH_2_), 33.5, 31.9, 31.8, 29.7 (8×C), 29.6 (2×C), 29.4, 26.0, 25.8, 22.7, 14.1; MS (ESI): 568 [M+H]^+^; Anal calcd for C_36_H_57_NO_4_: C, 76.15; H, 10.12; N, 2.47. Found C, 76.51; H, 9.74; N, 2.46. 

*(4S,5S)-4-Benzyloxy-5-hydroxy-N-(4-methoxybenzyl)-7-methyloctanoyl amide* (*syn*-**10h**): Waxy solid; [α]^25^_D_: -7.34 (*c* 2.9, CHCl_3_); IR (film) ν_max_: 3410, 3303, 1644, 1513, 1248 cm^-1^; ^1^H-NMR (400 MHz, CDCl_3_): δ 7.35-7.23 (m, 5H, Ar-H), 7.15 (d, *J* = 8.5 Hz, 2H, Ar-H), 6.85 (d, *J* = 8.5 Hz, 2H, Ar-H), 5.85 (s, 1H, NH), 4.59 (d, *J* = 11.5 Hz, 1H, OCH_2_), 4.53 (d, *J* = 11.5 Hz, 1H, OCH_2_), 4.33 (dd, *J* = 14.4, 5.8 Hz, 1H, NCH_2_), 4.27 (dd, *J* = 14.4, 5.8 Hz, 1H, NCH_2_), 3.78 (s, 3H, OCH_3_), 3.65-3.57 (m, 1H, H-4), 3.32 (m, 1H, H-5), 2.37 (d, *J* = 4.9 Hz, 1H, OH), 2.25 (t, *J* = 7.2 Hz, 2H, H-2), 2.08-1.98 (m, 1H), 1.93-1.75 (m, 2H), 1.48-1.38 (m, 1H), 1.27-1.18 (m, 1H), 0.92 (d, *J* = 6.7 Hz, 3H, CH_3_), 0.88 (d, *J* = 6.7 Hz, 3H, CH_3_); ^13^C-NMR (100 MHz, CDCl_3_): δ 172.4 (C=O), 158.9, 138.2, 130.3, 129.1 (2×C), 128.4 (2×C), 127.9 (2×C), 127.8, 114.0 (2×C), 81.6 (C-5), 72.6 (C-4), 70.6 (OCH_2_), 55.2 (OCH_3_), 43.0 (NCH_2_), 42.3, 31.8, 25.9, 24.5, 23.6, 21.7; MS (ESI): 400 [M+H]^+^, 422 [M+Na]^+^, 438 [M+K]^+^; Anal calcd for C_24_H_33_NO_4_: C, 72.15; H, 8.33; N, 3.51. Found C, 72.19; H, 8.16; N, 3.29. 

*(4S,5S)-4-Benzyloxy-5-hydroxy-N-(4-methoxybenzyl)-5-phenylpentanoyl amide* (*syn*-**10i**): White solid, mp: 45-47 °C; [α]^25^_D_: +14.09 (*c* 2.7, CHCl_3_); IR (film) ν_max_: 3411, 3307, 1655, 1512, 1249 cm^-1^; ^1^H- NMR (400 MHz, CDCl_3_): δ 7.36-7.23 (m, 10H, Ar-H), 7.15 (d, *J* = 8.6 Hz, 2H, Ar-H), 6.86 (d, *J* = 8.6 Hz, 2H, Ar-H), 5.52 (s, 1H, NH), 4.83 (d, *J* = 6.1 Hz, 1H, H-5), 4.51 (d, *J* = 11.5 Hz, 1H, OCH_2_), 4.46 (d, *J* = 11.5 Hz, 1H, OCH_2_), 4.28 (dd, *J* = 14.4, 5.6 Hz, 1H, NCH_2_), 4.22 (dd, *J* = 14.4, 5.6 Hz, 1H, NCH_2_), 3.77 (s, 3H, OCH_3_), 3.63 (ddd, *J* = 6.3, 6.1, 5.2 Hz, 1H, H-4), 3.06 (d, *J* = 4.0 Hz, 1H, OH), 2.13 (t, *J* = 7.6 Hz, 2H, H-2), 1.90 (m, 1H, H-3), 1.78 (m, 1H, H-3); ^13^C-NMR (100 MHz, CDCl_3_): δ 172.3 (C=O), 159.0, 141.1, 138.0, 130.4, 129.2 (2×C), 128.5 (2×C), 128.3 (2×C), 128.1 (2×C), 127.9 (2×C), 126.8 (2×C), 114.1 (2×C), 82.6 (C-5), 75.8 (C-4), 72.0 (OCH_2_), 55.3 (OCH_3_), 43.1 (NCH_2_), 32.0, 26.5; MS (ESI): 420 [M+H]^+^, 442 [M+Na]^+^, 458 [M+K]^+^; Anal calcd for C_26_H_29_NO_4_: C, 74.44; H, 6.97; N, 3.34. Found C, 74.49; H, 6.82; N, 3.59. 

*(4S,5S)-4-Benzyloxy-5-hydroxy-N-(4-methoxybenzyl)-6-phenylhexanoyl amide* (*syn*-**10j**): Waxy solid; [α]^25^_D_: +2.01 (*c* 2.6, CHCl_3_); IR (film) ν_max_: 3403, 3305, 1644, 1513, 1248, 1030 cm^-1^; ^1^H-NMR (400 MHz, CDCl_3_): δ 7.35-7.23 (m, 8H, Ar-H), 7.17-7.12 (m, 4H, Ar-H), 6.83 (d, *J* = 8.7 Hz, 2H, Ar-H), 5.55 (s, 1H, NH), 4.61 (d, *J* = 11.5 Hz, 1H, OCH_2_), 4.54 (d, *J* = 11.5 Hz, 1H, OCH_2_), 4.30 (dd, *J* = 14.5, 5.6 Hz, 1H, NCH_2_), 4.25 (dd, *J* = 14.5, 5.6 Hz, 1H, NCH_2_), 3.82-3.75 (m, 1H, H-4), 3.75 (s, 3H, OCH_3_), 3.39 (ddd, *J* = 5.8, 5.8, 5.1 Hz, 1H, H-5), 2.85 (dd, *J* = 13.8, 4.8 Hz, 1H, H-6), 2.75 (dd, *J* = 13.8, 8.3 Hz, 1H, H-6), 2.45 (d, *J* = 5.8 Hz, 1H, OH), 2.23 (t, *J* = 7.3 Hz, 2H, H-2), 2.07 (ddd, *J* = 14.0, 7.3, 5.6 Hz, 1H, H-3), 1.93 (ddd, *J* = 14.0, 7.3, 7.0 Hz, 1H, H-3); ^13^C-NMR (100 MHz, CDCl_3_): δ 172.4 (C=O), 158.9, 138.6, 138.2, 130.3, 129.3 (2×C), 129.1 (2×C), 128.4 (4×C), 128.0 (2×C), 127.8, 126.3, 114.0 (2×C), 79.8 (C-5), 73.6 (C-4), 72.2 (OCH_2_), 55.2 (OCH_3_), 43.0 (NCH_2_), 39.7, 31.9, 25.7; MS (ESI): 434 [M+H]^+^, 456 [M+Na]^+^, 472 [M+K]^+^; Anal calcd for C_27_H_31_NO_4_: C, 74.80; H, 7.21; N, 3.23. Found C, 74.83; H, 7.55; N, 3.28. 

*(4S,5S)-4-Benzyloxy-5-hydroxy-N-(4-methoxybenzyl)-7-phenylheptanoyl amide* (*syn*-**10k**): Waxy solid. [α]^25^_D_: -7.35 (*c* 1.9, CHCl_3_); IR (film) ν_max_: 3411, 3306 2932, 1645, 1513, 1248, 1030 cm^-1^; ^1^H-NMR (400 MHz, CDCl_3_): δ 7.40-7.20 (m, 10H, Ar-H), ,7.15 (d, *J* = 8.6 Hz, 2H, Ar-H), 6.85 (d, *J* = 8.6 Hz, ;2H, Ar-H), 5.63 (s, 1H, NH), 4.50 (d, *J* = 11.5 Hz, 1H, OCH_2_), 4.47 (d, *J* = 11.5 Hz, 1H, OCH_2_), 4.33 (dd, *J* = 14.5, 5.6 Hz, 1H, NCH_2_), 4.25 (dd, *J* = 14.5, 5.6 Hz, 1H, NCH_2_), 3.78 (s, 3H, OCH_3_), 3.71 (m, 1H, H-4), 3.37 (m, 1H, H-5), 2.90 (m, 2H), 2.67 (ddd, *J* = 13.8, 9.5, 7.0 Hz, 1H, H-3), 2.38 (d, *J* = 7.6 Hz, 2H, H-2), 2.52 (d, *J* = 5.6 Hz, 1H, OH), 2.08 (ddd, *J* = 13.8, 7.6, 5.2 Hz, 1H, H-3), 1.93-1.78 (m, 2H); ^13^C-NMR (100 MHz, CDCl_3_): δ 172.9 (C=O), 159.0, 142.1, 138.0, 130.2, 129.4, 129.2 (2×C), 128.5, 128.1 (4×C), 128.0 (2×C), 127.8, 125.5, 114.0 (2×C), 81.2 (C-5), 72.5 (C-4), 70.3 (OCH_2_), 55.1 (OCH_3_), 43.1 (NCH_2_), 34.9, 32.1, 31.7, 23.3; MS (ESI): 448 [M+H]^+^, 470 [M+Na]^+^; Anal calcd for C_28_H_33_NO_4_: C, 75.14; H, 7.43; N, 3.13. Found C, 75.23; H, 7.75; N, 3.39. 

### 4.3. The synthesis of (5S,6R)-2-Piperidinone ***8a*** via the cyclization of ***10a***

To a cooled (−20 °C) solution of a mixture of **10a** (182 mg, 0.51 mmol) and Et_3_N (0.14 mL, 1.00 mmol) in CH_2_Cl_2_ (5 mL) was added dropwise MsCl (0.047 mL, 0.61 mmol) under a nitrogen atmosphere. The mixture was stirred at −20 ~ −10 °C for 1 h. Water was added and the aqueous layer was separated and extracted with CH_2_Cl_2_. The combined organic layers were washed with brine, dried over anhydrous Na_2_SO_4_, filtered, and concentrated under reduced pressure. The residue was purified by flash chromatography on silica gel (eluent: EtOAc/P.E. = 1:2) to yield the mesylate **11** (202 mg), which is unstable and was used immediately in the next step. To a solution of mesylate** 11** (202 mg, 0.43 mmol) in THF (3 mL) and HMPA (0.15 mL, 0.86 mmol) was added dropwise a solution of potassium *tert*-butoxide (58 mg, 0.52 mmol) in THF (2 mL) at 0 °C under nitrogen atmosphere. The mixture was allowed slowly warming to room temperature and was stirred for 24 h. The reaction was quenched with saturated NH_4_Cl at 0 °C. The aqueous layer was separated and extracted with CH_2_Cl_2_. The combined organic layers were washed with brine, dried over anhydrous Na_2_SO_4_, filtered, and concentrated under reduced pressure. The residue was purified by flash chromatography on silica gel (eluent EtOAc/P.E. = 1:2) to yield (5*S*,6*R*)-**8a** (135 mg, 78% yield). For the data of (5*S*,6*R*)-**8a** see [20]. 
